# A Case of Post-traumatic Stress Disorder (PTSD) Complicated by Stockholm Syndrome: A Unique Psychiatric Phenomenon in the Context of Intimate Partner Violence

**DOI:** 10.7759/cureus.82307

**Published:** 2025-04-15

**Authors:** Noor Alshwaiheen, Deena Saleh, Waiel Alani

**Affiliations:** 1 Medicine and Surgery, Bahrain Defence Force Hospital, Riffa, BHR; 2 Psychiatry, Bahrain Defence Force Hospital, Riffa, BHR

**Keywords:** clinical psychiatry, intimate partner violence (ipv), psychiatry, ptsd (post-traumatic stress disorder), stockholm syndrome

## Abstract

Post-traumatic stress disorder (PTSD) and Stockholm syndrome are two distinct psychological responses that could occur following intense trauma. While PTSD is characterized by intrusive memories, hypervigilance, and avoidance behaviors, Stockholm syndrome involves the paradoxical development of emotional attachment to the abuser who inflicted the trauma. This case report presents a 39-year-old female patient who survived a life-threatening neck stabbing incident by her abusive ex-husband and developed both PTSD and Stockholm syndrome. Initially presenting with classic PTSD symptoms such as hyper-arousal, intrusive memories, and anxiety, the patient’s psychological recovery was complicated by a growing emotional attachment to her abuser. Over the course of treatment, her PTSD symptoms improved, but she expressed empathy for her attacker, maintained contact with him while he was incarcerated, and even sought to drop charges against him. This case highlights the complex interplay between PTSD and Stockholm syndrome, suggesting that PTSD symptom resolution may contribute to or even coincide with the development of Stockholm syndrome, particularly in cases of intimate partner violence (IPV). This report also recognizes the need for clinicians to recognize the potential emergence of Stockholm syndrome during PTSD recovery and to consider its implications for treatment and recovery in IPV cases.

## Introduction

Post-traumatic stress disorder (PTSD) is defined as a psychiatric disorder that can develop in individuals who have witnessed or experienced severe psychological trauma, emotional distress, or in reaction to physical injury, such as violent assaults, military combat, or other life-threatening events. Individuals with PTSD often experience intense and distressing thoughts and emotions in relation to their traumatic experience, which can persist long after the event or incident has occurred. [1]

In contrast to PTSD, Stockholm syndrome or “traumatic bonding” refers to a hypothetical, psychological phenomenon occurring in trauma survivors, characterized by feelings of trust, affection, and attachment toward their abuser. This psychological phenomenon was first observed in 1973 following a bank robbery in Stockholm, where hostages began to develop positive feelings toward their captors. It is believed that the bond is initially created when a captor threatens a captive’s life, deliberates, and then chooses not to kill or further harm the victim. The captive’s relief at the removal of the death threat is transposed into feelings of gratitude toward the captor for giving him or her life. These feelings of relief and gratitude are what create the paradoxical emotional attachment to the abuser. The term has been used in mainstream culture, legal contexts, and certain clinical settings, referring to the phenomenon where trauma survivors develop strong emotional attachments to their abusers. However, there is limited empirical research on the features of Stockholm syndrome; hence, there are no validated diagnostic criteria, and it is not recognized as a definitive psychiatric diagnosis [2-4].

This case highlights the intricate relationship between PTSD and Stockholm syndrome within the same individual. A female who experienced intimate partner violence (IPV), which resulted in a life-threatening neck stabbing incident. After enduring such a traumatic experience, she developed a range of emotional and psychological responses. Initially, she experienced feelings of fear and anxiety, but later on, she was found to have developed feelings of attachment toward her abuser. Notably, there is a significant paucity of documented cases illustrating the co-occurrence of PTSD and Stockholm syndrome in the same individual. Therefore, this case aims to explore the intersection between the two psychological responses to trauma, offering insights into their relationship and potential implications for treatment strategies. This case aims to provide valuable insight into the complexities of Stockholm syndrome in domestic violence scenarios and its relationship to PTSD, exploring whether the chronicity of PTSD or the absence of it might play a role in the paradoxical development of Stockholm syndrome.

## Case presentation

Background

A 39-year-old woman was referred for psychiatric evaluation following a life-threatening stabbing incident perpetrated by her abusive ex-husband. She had a history of two previous marriages and five children, of whom only two currently reside with her. Her past occupation included working under the Ministry of Interior in a prison where she had close contact with inmates. In 2020, she entered her third marriage with the individual responsible for this incident. Initially, their relationship appeared stable as he moved into her apartment with her and her two children. However, he began to exhibit increasing signs of jealousy, possessiveness, and emotional and financial dependence. The patient reported that he insisted on spending all his time with her and wanted to monitor her activities constantly due to unfounded doubts and fears of infidelity. His behavior was further aggravated by his alcohol consumption, which often led to verbal and physical abuse.

In early 2024, a fire broke out in the apartment, and the patient suspected that her partner had started it in a fit of anger. Although she lacked definitive evidence to support her claim, she took action by pressing charges and reporting the incident to the authorities, after which he left the apartment and did not reside with her anymore. In the aftermath, her partner began stalking her, frequently appearing outside her residence and trying to reach her through calls and texts. The patient was afraid to let him inside the apartment, yet she did accept his calls and maintained contact with him online.

A month later, the patient was preparing for work and had unlocked her apartment door to allow maintenance workers in. Unbeknownst to her, her partner entered the residence and launched an attack, stabbing her three times in the neck and head. The patient noted that he appeared to be clearly intoxicated during the incident and did not communicate verbally before, during, or after the attack. He simply left the scene afterward. The maintenance workers discovered her and promptly took her to the hospital. She was found to have a deep right temporal laceration, two deep stab wounds in the neck with diffused hematoma, as well as bruises on her lower limbs. Further imaging with CT showed a contrast leak with deviation of neck vessels to the right side caused by a subcutaneous left-sided hematoma. She required admission to the ICU and underwent three surgeries (pseudoaneurysm stenting), which left her with long-term complications, including left eye ptosis, post-traumatic Horner’s syndrome, and intermittent headaches with photophobia. Following her medical recovery, psychiatry was consulted to address the patient’s mental health in the aftermath of this traumatic event.

Psychiatric presentation

Following the incident, the patient exhibited symptoms consistent with the PTSD diagnostic criteria, such as intense anxiety, insomnia, flashbacks, and hypervigilance. She developed avoidance behaviors, reacting with heightened emotional responses to words such as "knife," "stabbing," and "prison." In an effort to safeguard herself from her now ex-husband, she would lock all the doors in her apartment, despite knowing that he was in prison and couldn’t reach her. The patient also experienced nightmares that kept her up at night and was initially reluctant to discuss the traumatic event in detail.

Treatment course

The treatment for this patient’s diagnosed PTSD began with pharmacological interventions, including lorazepam 1 mg tablet as needed for anxiety management. Paroxetine XR tablet was initiated at a dosage of 12.5 mg daily, which was subsequently titrated to 25 mg after two weeks. Additionally, mirtazapine 30 mg tablet was prescribed in conjunction with paroxetine to augment its effects in regard to the PTSD symptoms.

In conjunction with the medications, she was referred to trauma-focused psychotherapy sessions. During her first session, she reported experiencing thoughts that she found perplexing. Despite the clear signs and symptoms of PTSD she was having, she also expressed sympathy toward her attacker, stating that she did not harbor any feelings of hatred toward him. She articulated that these feelings were confusing and causing her emotional turmoil as she struggled to understand the reason behind her emotional responses or how to manage them effectively. Although she was not following up regularly for the psychotherapy sessions, it was evident that she began showing early signs of Stockholm syndrome. This was further made apparent by her having reservations about pursuing legal charges against her ex-husband.

Over the course of five to six months on medications, her PTSD symptoms improved; however, the growing empathy toward her abuser grew stronger and was more obvious. She became increasingly concerned for his well-being and began to minimize the severity of his assault on her.

During one of her follow-up appointments, she conveyed strong feelings of empathy toward her ex-partner and disclosed that she has been maintaining regular contact with him through phone calls while he is in prison. She expressed her belief that he has “gotten better” with the assistance of the prison psychiatrist. Additionally, she confirmed that her decision to drop the charges against him is definitive, sharing that she feels significantly better after reaching that conclusion. This decision was influenced by her perspective that his initial 15-year sentence was excessively long and that he does not deserve to spend such an extended time behind bars. This shift in her emotional attachment toward her abuser strongly indicates the presence of signs and symptoms consistent with Stockholm syndrome.

## Discussion

Stockholm syndrome and PTSD: a paradoxical interplay

This case highlights the complex and contradictory relationship between PTSD and Stockholm syndrome. The patient, a victim of IPV who experienced a life-threatening stabbing incident by her abusive ex-husband exhibited classical symptoms of PTSD following the attack. Her symptoms of intrusive recollections of the incident, severe anxiety, hyper-arousal, insomnia, and flashbacks fit the typical picture of PTSD, where the symptoms fall into three distinct clusters: intrusive recollections, avoidant/numbing symptoms, and hyper-arousal symptoms. These symptoms in direct response to the traumatic attack, the duration being more than one month, and the symptoms leading to significant distress further confirm the diagnosis of PTSD. However, as her PTSD symptoms began to improve over the course of treatment, a paradoxical psychological response emerged, marked by the development of empathy and attachment toward her abuser. This shift in emotional response is characteristic of Stockholm syndrome, where there is a complex psychological development of positive feelings toward her abuser. In this case, the patient may have felt a sense of relief at surviving the abusive relationship and subsequent violent attack, which may have led to feelings of gratitude and attachment toward her abuser, despite the life-threatening incident she endured [4-5].

The development of Stockholm syndrome

Stockholm syndrome, while not officially classified as a psychiatric diagnosis, has been well-described in the literature as a response to extreme and prolonged trauma, particularly in situations involving IPV and captivity. This particular phenomenon has drawn much attention from the media in recent years, being highlighted more prominently in various kidnapping cases such as Patty Hearst (1974), Elizabeth Smart (2002), and Jaycee Dugard (2009). For the patient in this case, her attachment to her abuser, despite the life-threatening violence he inflicted toward her, can be seen through the lens of a survival-based coping mechanism [2,6-7].

There is ongoing debate among experts regarding individual susceptibility to Stockholm syndrome, with some asserting that certain individuals may be more prone to developing it than others. Additionally, there is no consensus on the defining characteristics of the syndrome. Since the initial case in 1973, researchers have examined similar occurrences in various contexts, including hostage situations, kidnappings, and cases of domestic violence. However, the diversity of these cases, differing in factors such as age, gender, location, number of individuals involved, type of abuse, and duration, complicates the establishment of a standardized set of diagnostic criteria. Despite these variations, most experts identify three core characteristics of Stockholm syndrome: first, hostages tend to develop negative perceptions of law enforcement or authorities; second, they form positive emotional bonds with their captors; and third, the captors, in turn, develop positive feelings toward their hostages [7].

For the patient in this case, as her PTSD symptoms began to diminish with the aid of pharmacotherapy and trauma-based psychotherapy, she began to express feelings of empathy toward her abuser. This marked deviation from her initial trauma response illustrates the co-occurrence of PTSD and Stockholm syndrome in the same individual. The underlying complexity of trauma responses suggests that PTSD symptom resolution may contribute to or even coincide with the development of Stockholm syndrome in certain contexts. Several hypotheses have been proposed to explain the phenomenon of Stockholm syndrome, with the most widely accepted theory coming from evolutionary psychologists. They suggest that this phenomenon is an inherited survival mechanism dating back to hunter-gatherer societies, where women would form bonds with members of rival tribes to increase their chances of survival. Stockholm syndrome is believed to develop in response to specific conditions, including perceived threats to one's life, the inability to escape, and small acts of kindness from captors. As a result, victims may exhibit symptoms such as positive feelings toward their abductors and a tendency to justify or support their abuser's actions [8].

Limited literature review of the relationship between PTSD and Stockholm syndrome has suggested that emotional attachment to an abuser is thought of as a maladaptive coping strategy. Furthermore, this “traumatic bonding” increases the likelihood of the victim being stuck in the abusive relationship, furthering the attachment and increasing the risk of developing PTSD symptoms. Put simply, while traumatic bonding may serve as an adaptive mechanism enabling IPV victims to endure the abusive relationship, it remains an unhealthy and maladaptive coping strategy as it can perpetuate their entrapment in such relationships. This cycle of abuse and feeling of being “trapped” might be a trigger point for later developing an emotional attachment to the abuser and exhibiting signs of Stockholm syndrome. However, other studies have found no significant association between PTSD and Stockholm syndrome in cases involving specifically kidnap victims. These differing hypotheses on the relationship between PTSD and Stockholm syndrome highlight the need for further research to fully understand the link, if any, between these two complex psychological responses to trauma [6].

The role of trauma resolution

While PTSD symptoms typically emerge immediately following a traumatic event and persist for weeks to months, Stockholm syndrome may develop over time, potentially serving as a coping mechanism to prolonged trauma. The timeline of symptom progression in this patient is significant, as after months of treatment for PTSD, her symptoms began to subside, and her attachment to her abuser grew stronger. However, since she began showing empathy toward her abuser from the first psychotherapy session, this could mean that the symptoms of Stockholm syndrome developed alongside her PTSD symptoms but were too complex for her to grasp; hence, they were not as evident. She began voicing concerns for her ex-husband and showing an emotional attachment to him, although not understanding why she felt that way. It was apparent that despite the fear she felt following the attack, she still did not despise her attacker. It is worth noting that the chronicity of PTSD symptoms, coupled with her past occupation of being close to other inmates and continued emotional engagement with her abuser, maintaining contact even while he was imprisoned, and being aware of his well-being, may have facilitated the progression of Stockholm syndrome. In this context, one must consider the psychological mechanisms at play, including the victim's efforts to comprehend the trauma or to regain a sense of control in an otherwise uncontrollable situation [9].

Theoretical insights and clinical implications

The relationship between PTSD and Stockholm syndrome offers valuable insights into the complexities of trauma recovery, especially in the context of IPV. As PTSD is characterized by constant anxiety, intrusive memories, and hyper-arousal, the transition to Stockholm syndrome signifies a shift from an acute trauma response to a more chronic adaptation to trauma. Over time, the victim may also begin to rationalize the abuse and “forgive” the abuser for what they have done. Psychologically, this can be seen as a coping mechanism for the overwhelming stress induced by the attack and perceived lack of control over the traumatic experience [6].

For clinicians, it is essential to understand that while PTSD treatments such as trauma-focused cognitive behavioral therapy and pharmacotherapy can effectively alleviate symptoms like anxiety and hyperarousal, this case questions if they inadvertently contribute to the development of Stockholm syndrome if the emotional connection to the abuser is not addressed early on. The relief from the PTSD symptoms might facilitate an emotional re-engagement with the abuser, which complicates the recovery process. Therefore, trauma-informed care must be attentive to the emergence of ambivalent feelings toward abusers, particularly in cases of IPV where the dynamics of abuse, love, and fear are deeply intertwined. Research has shown that the treatment for Stockholm syndrome closely resembles that of PTSD, often involving family therapy, group therapy, and counseling to help individuals process their experiences. Ultimately, Stockholm syndrome functions as a psychological defense mechanism, allowing victims to cope with extreme stress and fear by forming an emotional attachment to their abuser. This attachment fosters the belief that bonding with the captor enhances their chances of survival, with even minor acts of kindness reinforcing a more favorable perception of the abuser [8].

Future research directions

This case emphasizes the necessity for additional research into the relationship between PTSD and Stockholm syndrome. Specifically, an exploration of the duration between the resolution of PTSD symptoms and the potential onset of Stockholm syndrome, as well as the factors that might contribute to this paradoxical attachment. Furthermore, it raises questions about the diagnostic criteria for Stockholm syndrome and whether it should be formally classified as a clinical diagnosis, given its considerable implications for survivors of IPV. Gaining a deeper understanding of the progression from PTSD to Stockholm syndrome could yield vital insights into trauma recovery and aid in the development of more tailored therapeutic interventions aimed at breaking the cycle of emotional attachment to the abuser.

## Conclusions

This case underscores the complexity of trauma responses, particularly in the context of IPV, where both PTSD and Stockholm syndrome can coexist and interact. The patient’s development of Stockholm syndrome, despite the life-threatening nature of the attack, suggests that PTSD symptom resolution might create a psychological environment conducive to the emergence of trauma bonding. This paradoxical emotional attachment could potentially undermine recovery, especially if the psychological shift toward empathy and attachment to the abuser is not addressed in therapy. Clinicians should be mindful of the potential for Stockholm syndrome to develop during the course of PTSD treatment, as patients may not only process their trauma but also begin to form positive emotional connections to their abusers. Further research is needed to better understand the relationship between PTSD resolution and the onset of Stockholm syndrome, as well as to establish clinical guidelines for managing these complex dynamics in trauma survivors. The development of tailored therapeutic approaches that address both the resolution of PTSD and the prevention of maladaptive emotional attachments to abusers could improve outcomes for survivors of IPV and other forms of extreme trauma.
